# The Effects of 12 Weeks of a Combined Exercise Program on Physical Function and Hormonal Status in Elderly Korean Women

**DOI:** 10.3390/ijerph16214196

**Published:** 2019-10-30

**Authors:** Ji Yu Im, Hyun Seok Bang, Dae Yun Seo

**Affiliations:** 1 Department of Physical Education, Chang Won National University, Chang Won 51140, Korea; ikmcool@naver.com; 2 Department of Physical Education, College of Health, Social Welfare and Education, Tong Myong University, Busan 48520, Korea; banghs@tu.ac.kr; 3 College of Medicine, Cardiovascular and Metabolic Disease Center, Inje University, Busan 47392, Korea

**Keywords:** elderly women, combined exercise, growth hormone, dehydroepiandrosterone sulfate, estrogen

## Abstract

Aging causes a decline in physical function and hormonal balance. Exercise can improve these parameters. However, the beneficial effects of a combined exercise program (Korean dance and yoga) on physical function and hormonal status in elderly women remain unknown. This study aims to investigate the effects of a 12-week combined exercise program on balance, flexibility, muscle strength, and hormonal status in elderly Korean women. Twenty-five healthy elderly women were recruited and randomly divided into the control (CON) and exercise (EXE) groups. The EXE group underwent the combined exercise program (60 min/day and 3 times/week) for 12 weeks. The two groups did not differ in body weight, lean body mass, fat mass, body fat percentage, or body mass index at baseline or in the changes following the experimental conditions. A significant time × group interaction was detected for anterior and posterior dynamic balance, static balance, and growth hormone (GH). After the combined exercise program, anterior dynamic balance, posterior dynamic balance, static balance, flexibility, muscle strength, GH, dehydroepiandrosterone-sulfate, and estrogen significantly increased in the EXE group compared to the CON group. In conclusion, the combined exercise program contributed to improvements in overall health, including physical function and hormonal status, in elderly Korean women.

## 1. Introduction

The elderly population aged >65 years accounted for 14.3% of the total Korean population in 2018 [1], and it is estimated to increase further from 24.3% in 2030 to 40.1% in 2060 [2]. Aging contributes to the multifactorial process that causes decline in physical activity levels, resulting in metabolic dysfunction and unbalanced hormonal status [3,4]. The increasing health costs due to aging urgently need to be addressed [5]. Thus, the maintenance of healthy aging by performing physical activity, promoting self-sufficiency, and providing leisure time becomes an important public health issue for improving an individual’s strength.

Variations in physical function and balanced hormonal status are among the important determinants of health and functional capacity in elderly women and men [6,7]. In particular, postmenopausal elderly women show decreased balance, resulting in falling accidents which cause muscle injuries, fractures, and neuromuscular dysfunctions [8,9]. Flexibility, defined as the range of motion of a given joint, decreases by up to 50% during aging [10]. Elderly women experience a decline in flexibility and muscle strength, which compromises the physical activities of daily living [11]. Therefore, improving these parameters is crucial in healthy elderly women.

Aging coupled with a sedentary lifestyle is accompanied by progressive decline in muscle strength, growth hormone (GH), dehydroepiandrosterone-sulfate (DHEA-S), and estrogen, which results in the rapid loss of skeletal muscle mass, greater fall injuries, impaired walking ability, and poor quality of life of elderly women [3,12,13]. GH is a peptide hormone that stimulates growth and cell reproduction and is stimulated by sex hormones such as testosterone, DHEA-S, and estrogen [14]; these regulate human development [15]. Decreases in GH contribute to the reduction of lean body mass and muscle strength as well as to increased body fat with aging [16]. Estrogen, the primary female sex hormone, is considered to play a significant role in women’s mental health as well as in the maintenance of bone mineral density and immune function [17]. DHEA-S is an endogenously produced sex steroid associated with anti-aging effects [18]. Decline in estrogen levels with aging is associated with reduced muscle strength in elderly women [19]. In elderly men, decline in DHEA-S levels is found to begin at the age of 65 years [20] and causes muscle weakness [21]. In elderly women, DHEA-S levels are negatively correlated with aging [22,23]. Therefore, the clinical meaning of DHEA-S levels in elderly women remains unclear.

Exercise programs are widely recommended to ameliorate aging-associated dysfunction through improvement in physical function and hormonal balance because of their low cost and non-pharmacological strategy [24]. Exercise is a useful tool for improving physical function, inhibiting hormonal deficiency, and maintaining the optimal quality of life in elderly women [25,26,27,28]. Thus, an exercise program is one of the most effective activities for the aging population. In Korea, local community exercise programs include a Korean dance that is one of the most popular physical activities for elderly women [29]. The Korean dance involves moving the limbs to traditional music [30]. Therefore, interventions based on traditional Korean dance represent an attractive therapeutic strategy to improve body composition in elderly women [31]. For example, the Korean dance can improve balance and attenuate falls and medical costs, suggesting that this program promotes healthy life and prevents fall accidents in the elderly [32]. Kim et al. [33] investigated the effect of the Korean dance on health-related fitness and blood lipids in elderly Korean women who participated in a Korean dance program for 12 weeks. They reported a significant improvement in grip strength and high-density lipoprotein cholesterol levels. These results suggest that the Korean dance is associated with a dynamic exercise program. In other studies, 12 weeks of a yoga exercise program, which is a representative static exercise, showed a possible beneficial effect in maintaining GH and DHEA-S, thus promoting healthy aging [34]. Alves et al. [35] reported that 12 weeks of a yoga exercise program improved flexibility, balance, coordination, and strength levels [36]. These factors contribute to the prevention of falls and improvement of higher physical activities of daily living [37]. Taken together, the Korean dance and yoga programs have a beneficial effect on physical function and hormonal status for elderly adults. However, the effects of the combined Korean dance and yoga exercise program for healthy aging remain unknown.

This study aimed to evaluate the effects of a 12-week combined exercise program on balance, flexibility, muscle strength, GH, DHEA-S, and estrogen in healthy elderly women. We hypothesized that the combined exercise program might improve balance, flexibility, muscle strength, GH, DHEA-S, and estrogen in this population.

## 2. Materials and Methods

### 2.1. Ethics Approval and Consent to Participate

The study was approved by the Chang Won University’s Ethics Committee (1040271–201802–HR–004). All procedures were performed in accordance with the Declaration of Helsinki for research on human subjects. All participants provided informed consent prior to participation.

### 2.2. Participants and Study Design

**Figure 1:**
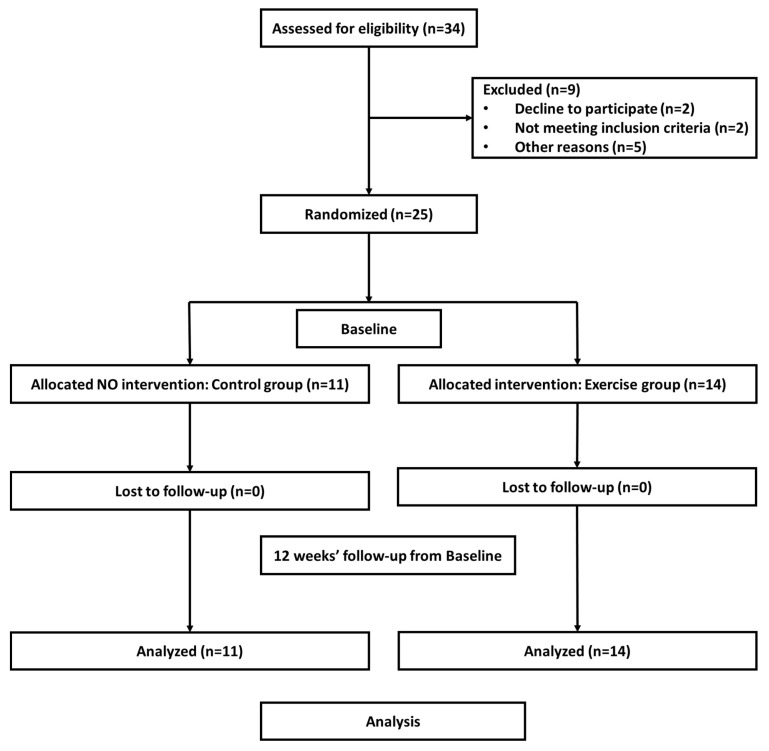
Flow diagram of the study.

A total of 34 Korean women, who were post-menopausal for at least five years, were recruited from a local community fitness center and voluntarily participated in the study. Four and five participants were excluded due to health issues and non-completion of the study intervention, respectively. Subsequently, the participants were randomly assigned to the following two groups at baseline: control group (CON, n = 11, mean age 69.36 ± 2.94 years) and exercise group (EXE, n = 14, mean age 71.57 ± 3.22 years). The flow chart of the selection process is shown in Figure 1. The baseline characteristics of the subjects are presented in Table 1.

The inclusion criteria were as follows: age ≥65 years, never having attended an exercise program, and had not participated in yoga or Korean dance lessons for the six months prior to the study. The exclusion criteria included a history of medical problems such as cardiovascular disease, musculoskeletal limitations, obesity, diabetes, and brain-related disease. In addition, the participants could not be taking any medication for hormonal and metabolic dysfunction. Balance, flexibility, muscle strength, and blood samples were evaluated at baseline (week 0) and post-exercise intervention (week 12). All data were collected at the same time of day (9:00 a.m.) after a day of performing the final combined exercise program and fasting for 12 h. The EXE (n = 14) group performed an exercise program (at 10:00 a.m., 60 min/day and 3 times/week ) that included warming up (5 min), the main exercise (50 min), and cool down (5 min) for 12 weeks. The combined exercise program included yoga and Korean dance for 12 weeks. The CON group (n = 11) did not perform any exercise program, but were supervised in the fitness center at the same frequency and time, and performed sedentary physical activities, such as listening to music, reading a book, and watching TV. The CON and EXE groups were strictly advised to maintain their regular physical activity and dietary habits during the intervention. All measurements and exercises were performed using standardized protocols with two expert investigators.

### 2.3. Perceived Exertion

The rating of perceived exertion (RPE) was assessed using the 20-point Borg scale of perceived exertion (6 = extremely easy to 20 = extremely hard) [38]. A participant’s RPE was assessed during each exercise intervention by supervisors.

### 2.4. Exercise Program

Each participant performed a supervised combined exercise program (yoga and Korean dance) for 60 min per day, 3 times per week with an RPE of 12–13. The program is shown in Table 2. The combined exercise program was supervised by a professional expert, and none of the participants performed any other exercise program during the study period.

### 2.5. Assessment of Body Composition, Balance, Flexibility, and Muscle Strength

*Body composition.* The participants arrived at the venue and were given a 30 min break. Then, their body composition was evaluated using bioelectrical impedance analysis (BIA) (InBody 520, Biospace Co., Seoul, Korea). We obtained height (cm), body weight (kg), body fat mass (FM) (kg), lean body mass (LBM) (kg), and body mass index (BMI—calculated as body mass divided by the height squared (kg/m^2^)) pre- and post-intervention.

*Static balance and dynamic balance ability.* Balance was measured using the Center of Pressure measuring method of the Humac Norm Balance System (Computer Sports Medicine Inc., Boston, MA, USA). The protocols were performed according to a previous study [39].

*Flexibility.* Participants performed the chair sit-and-reach test according to the senior fitness test protocol as described previously [40]. The participants had three trial attempts and the highest score was recorded.

*Muscle strength.* Participants performed the chair stand test according to the senior fitness test protocol as described previously [40].

### 2.6. Blood Collection and Measurements

Blood samples were obtained from the vein at baseline (week 0) and post intervention (week 12) after 12 h of fasting. The blood was collected in serum-separating tubes, centrifuged for 10 min at 1977 × *g* to obtain the serum without other components of blood, and immediately frozen at −80 °C until analyzed. GH level was measured using a chemiluminescence immunoassay method (Siemens, Los Angeles, CA, USA). DHEA-S level was measured by performing the electrochemiluminescence immunoassay method (Roche Diagnostics GmbH, Mannheim, Germany), and estrogen level was measured using the radioimmunoassay method (Perkin Elmer, Boston, MA, USA).

### 2.7. Statistics

All data are presented as means ± standard deviation. To determine data normality, we used the Shapiro–Wilk test. A two-way analysis of variance (ANOVA) with repeated measures [group (CON and EXE) × time (baseline and post-12 weeks)] was used to determine the difference in the changes in the dependent variables between baseline and post-combined exercise programs within and between groups with a post-hoc Bonferroni test. When a significant interaction was noted, paired *t*-tests were used for post-hoc comparisons. The level of significance was set to *p* < 0.05. Student’s *t*-test (unpaired) (post–baseline) was used to determine the changes in the variables and the difference between groups for change (Δ).

## 3. Results

Table 2 shows the demographic characteristics of the participants at baseline and 12 weeks after the two experimental conditions. At baseline, there were no differences in age, height, weight, LBM, FM, BF, and BMI between the groups. No differences were found in body weight, LBM, FM, BF percentage, and BMI between baseline and after the two experimental conditions in both groups (Table 2).

**Figure 2:**
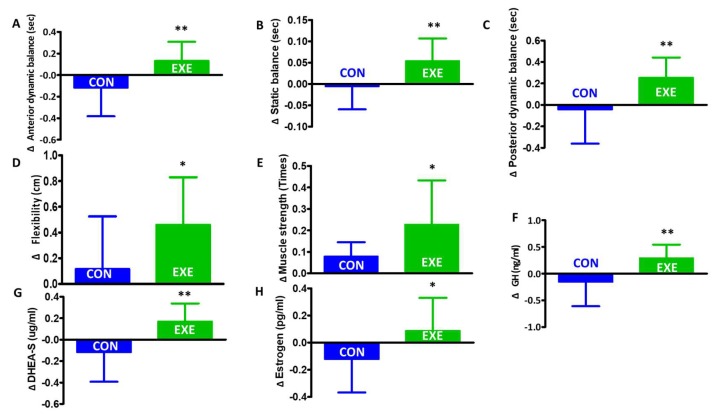
Changes in anterior dynamic balance, posterior dynamic balance, static balance, flexibility, muscle strength, growth hormone (GH), dehydroepiandrosterone-sulfate (DHEA-S), and estrogen from baseline to 12-weeks post-intervention in the control (CON) or exercise (EXE) group. * *p* < 0.05 compared to CON, ** *p* < 0.01 compared to CON. Values are presented as mean ± SD. (**A**) anterior dynamic balance (*p* = 0.009), (**B**) posterior dynamic balance (*p* = 0.008), (**C**) static balance (*p* = 0.010), (**D**) flexibility (*p* = 0.040), (**E**) muscle strength (*p* = 0.030), (**F**) GH (*p* = 0.040), (**G**) DHEA-S (*p* = 0.003), and (**H**) estrogen (*p* = 0.046).

A significant time × group interaction was detected for anterior (*p* = 0.016) and posterior dynamic balance (*p* = 0.032), static balance (*p* = 0.003), and GH (*p* = 0.037) (Table 3). After the combined exercise program, in the EXE group, anterior dynamic balance (*p* = 0.009), posterior dynamic balance (*p* = 0.008), static balance (*p* = 0.010), flexibility (*p* = 0.040), muscle strength (*p* = 0.030), GH (*p* = 0.040), DHEA-S (*p* = 0.003), and estrogen (*p* = 0.046) significantly increased compared to those of the CON group (Figure 2A–H).

## 4. Discussion

To the best of our knowledge, this is the first study investigating the effect of a combined exercise program on anterior dynamic balance, posterior dynamic balance, static balance, flexibility, muscle strength, GH, DHEA-S, and estrogen in elderly Korean women. The main finding of this study is that there was a significant improvement in anterior dynamic balance, posterior dynamic balance, static balance, flexibility, muscle strength as well as in GH, DHEA-S, and estrogen levels following training in elderly Korean women.

Aging is associated with a decline in physical function (i.e., balance, flexibility, and muscle strength) and hormonal status (i.e., GH, DHEA-S, and estrogen) [34,41,42,43,44]. Previous studies have also reported that an exercise program improves physical function and prevents hormonal deficiency in elderly women [45,46]. However, the effects of a combined exercise program that includes Korean dance, which is a dynamic exercise, and yoga, which is a static exercise, on these parameters have not yet been reported. In this study, we tested whether a moderately intense combined Korean dance and yoga exercise program may contribute to increased balance, flexibility, muscle strength, GH, DHEA-S, and estrogen levels. We found a significantly greater increase in balance, flexibility, and muscle strength following the combined exercise program compared to the control group. The findings of our study suggest that increased balance, flexibility, and muscle strength reduce fall occurrence and improve optimal quality of life in elderly women. These observations are consistent with those of a previous study that reported an increase in balance and flexibility following a yoga exercise program [47,48]. It is also possible that Korean dance is associated with an increase in muscular strength [33]. Taken together, these results may lead to a decline of fall incidents among the elderly [49,50,51].

Aging is related to a decline in the endocrine system. In recent years, accumulating evidence suggests the therapeutic role of GH, DHEA-S, and estrogen in elderly women [52,53,54]. A deficiency of GH during aging increases adiposity and decreases LBM [55]. Another study reported that administration of GH improved the body composition, such as FM and LBM, suggesting GH administration as an anti-aging treatment [56,57]. However, a previous study suggested that GH treatment may cause various side effects, such as joint pain and edema [58,59]. To avoid these side effects, we studied the beneficial effects of the combined exercise program—which is suggested to ameliorate the side effects—on body composition. In the present study, we found that the combined exercise program induced an improvement of GH, and increased GH was not associated with improvement in body composition. These findings are congruent with those of previous investigations demonstrating that an increase in GH through combined exercises is not associated with muscle mass and strength [58].

Fukai et al. [60] demonstrated that a higher DHEA-S level contributes to the improvement in physical activity levels and activities of daily living in elderly women. Other studies reported that low DHEA-S levels are associated with depressive symptoms and cognitive impairment in aging people [61,62]. It is necessary to study a therapeutic strategy to normalize and improve DHEA-S levels. In this study, we tested whether the combined exercise program played a role in regulating DHEA-S levels. We found that this exercise program increased DHEA-S levels, suggesting that this therapeutic exercise program may address depressive symptoms and cognitive impairment in elderly women. Reduction in estrogen levels due to aging contributes to the loss of LBM, resulting in unhealthy lifestyle [63]. In terms of the effects of estrogen levels after the combined exercise program, we found that the EXE group did not show improvement in LBM compared to the CON group, but we found a significant increase in estrogen levels in the EXE group, which was in line with the results of a previous study by Chun et al. [64], who found that a combined exercise program was effective in improving estrogen levels. Keriie et al. [65] suggested that 12 weeks of endurance exercise training increased estrogen levels in postmenopausal women. Taken together, these results support the assumption that a combined exercise program could be a new therapeutic tool for improving the hormonal status of elderly women.

The current study has several limitations. First, it included a small sample size (n = 25). Second, the participants were limited to elderly women (age = 65 years). Third, it was not possible to evaluate the isolated effect of the exercise protocol; hence, we could not provide the beneficial effect of each exercise program (Korean dance alone or yoga alone). In the future, it will be necessary to conduct a study involving a larger sample size with participants of different ages. Moreover, the effects of each exercise intervention should be separately investigated.

## 5. Conclusions

The 12-week combined exercise program significantly increased the anterior dynamic balance, posterior dynamic balance, static balance, flexibility, muscle strength, GH, DHEA-S, and estrogen levels of elderly Korean women. These findings provide evidence that a combined exercise program may serve as a new exercise strategy for healthy aging among Korean women.

## Figures and Tables

**Table 1:** Description of the combined exercise program performed by the exercise group.

	Exercise		Duration	Frequency	Week	Intensity
Warm up	Stretching		5 min	3 times/week	1–6	RPE 12
Main exercise	Static balance exercise	Valdidim, Parlsawee, Zainngareem, Saetaryeong	30 min			
	Dynamic balance exercise	A sitting position, Sitting Forward Bend Pose, Butterfly Pose, Bat Pose, God of the Half Fishes Pose, Cow Facing Pose	20 min		6–12	RPE 13
Cool-down	Stretching, respiration arrangement, and meditation (Baddha Konasana)		5 min			

RPE: rating of perceived exertion.

**Table 2:** Demographic characteristics at baseline and 12 weeks in the control and exercise groups.

Variable ^1^	Intervention				Effects (*p*-Value)		
	Control Group (n = 11)		Exercise Group (n = 14)				
	Baseline	12 Weeks	Baseline	12 Weeks	Time	Group	Time × Group
Age (years)	69.36 ± 2.94	-	71.57 ± 3.22	-	N.A.	N.A.	N.A.
Height (cm)	154.63 ± 5.04	-	153.57 ± 7.11	-	N.A.	N.A.	N.A.
Weight (kg)	62.83 ± 7.58	63.00 ± 8.00	58.76 ± 9.01	59.32 ± 8.94	0.877	0.116	0.936
LBM (kg)	39.40 ± 4.39	38.87 ± 4.53	37.02 ± 4.50	37.17 ± 4.39	0.877	0.115	0.790
FM (kg)	23.41 ± 3.98	24.13 ± 4.47	21.72 ± 5.52	22.15 ± 5.30	0.685	0.199	0.918
BF (%)	37.19 ± 2.93	38.16 ± 3.46	36.58 ± 4.73	36.99 ± 4.08	0.542	0.433	0.802
BMI (kg.m^−2^)	26.36 ± 2.38	26.31 ± 2.73	24.77 ± 2.84	25.07 ± 2.37	0.868	0.062	0.820

^1^ LBM: lean body mass; FM: fat mass; BF: body fat percentage; BMI: body mass index; N.A.: non-applicable.

**Table 3:** Participants’ anterior dynamic balance, posterior dynamic balance, static balance, flexibility, muscle strength, growth hormone (GH), dehydroepiandrosterone-sulfate (DHEA-S), and estrogen at baseline and 12 weeks post-intervention in the control or exercise group.

Variable	Intervention				Effects (*p*-Value)		
	Control Group (n = 11)		Exercise Group (n = 14)				
	Baseline	12 Weeks	Baseline	12 Weeks	Time	Group	Time × Group
Anterior dynamic balance (sec)	32.18 ± 3.81	30.09 ± 6.90	32.35 ± 5.34	37.78 ± 4.59	0.270	0.012	0.016
Posterior dynamic balance (sec)	22.27 ± 4.62	22.81 ± 7.01	21.64 ± 5.10	29.71 ± 6.75	0.15	0.072	0.032
Static balance (sec)	87.18 ± 3.60	86.90 ± 2.77	86.71 ± 3.60	91.71 ± 1.85	0.008	0.014	0.003
Flexibility (cm)	11.36 ± 9.40	13.60 ± 9.00	10.57 ± 8.59	18.11 ± 7.49	0.51	0.452	0.284
Muscle strength	12.72 ± 3.63	13.72 ± 3.28	14.35 ± 3.05	19.35 ± 4.60	0.007	0.001	0.066
GH (ng/mL)	1.35 ± 0.41	1.24 ± 0.33	1.14 ± 0.46	1.88 ± 1.11	0.109	0.278	0.037
DHEA-S (µg/mL)	46.89 ± 20.01	43.30 ± 19.38	62.15 ± 33.83	75.90 ± 40.84	0.568	0.010	0.332
Estrogen (pg/mL)	26.36 ± 2.38	26.31 ± 2.73	24.77 ± 2.84	25.07 ± 2.37	0.868	0.062	0.820
